# Bone Morphogenetic Protein (BMP)-7 expression is decreased in human hypertensive nephrosclerosis

**DOI:** 10.1186/1471-2369-11-31

**Published:** 2010-11-16

**Authors:** Carsten P Bramlage, Björn Tampe, Michael Koziolek, Imad Maatouk, Jelena Bevanda, Peter Bramlage, Katharina Ahrens, Katharina Lange, Holger Schmid, Clemens D Cohen, Matthias Kretzler, Gerhard A Müller

**Affiliations:** 1 Department of Medicine, Nephrology and Rheumatology, Georg-August-University Göttingen, Robert-Koch-Strasse 40, 37075 Göttingen, Germany; 2 Institute for Cardiovascular Pharmacology and Epidemiology, Menzelstrasse 21, 15831 Mahlow, Germany; 3 Department of Medical Statistics, Georg-August-University Göttingen, Robert-Koch-Strasse 40, 37075 Göttingen, Germany; 4 Department of Nephrology, Medical Policlinic, Ludwig Maximilians University Munich, Pettenkoferstr. 8a, D - 80336 Munich, Germany; 5 Division of Nephrology and Institute of Physiology, University Zürich, Rämistr. 100, 8091 Zürich, Switzerland; 6 Internal Medicine - Nephrology, University of Michigan, 1150 W. Medical Centre, Ann Arbor, USA

## Abstract

**Background:**

Bone Morphogenetic Protein (BMP)-7 is protective in different animal models of acute and chronic kidney disease. Its role in human kidneys, and in particular hypertensive nephrosclerosis, has thus far not been described.

**Methods:**

BMP-7 mRNA was quantified using real-time PCR and localised by immunostaining in tissue samples from normal and nephrosclerotic human kidneys. The impact of angiotensin (AT)-II and the AT-II receptor antagonist telmisartan on BMP-7 mRNA levels and phosphorylated Smad 1/5/8 (pSmad 1/5/8) expression was quantified in proximal tubular cells (HK-2). Functional characteristics of BMP-7 were evaluated by testing its influence on TGF-β induced epithelial-to-mesenchymal transition (EMT), expression of TGF-β receptor type I (TGF-βRI) and phosphorylated Smad 2 (pSmad 2) as well as on TNF-α induced apoptosis of proximal tubular cells.

**Results:**

BMP-7 was predominantly found in the epithelia of the distal tubule and the collecting duct and was less abundant in proximal tubular cells. In sclerotic kidneys, BMP-7 was significantly decreased as demonstrated by real-time PCR and immunostaining. AT-II stimulation in HK-2 cells led to a significant decrease of BMP-7 and pSmad 1/5/8, which was partially ameliorated upon co-incubation with telmisartan. Only high concentrations of BMP-7 (100 ng/ml) were able to reverse TNF-α-induced apoptosis and TGF-β-induced EMT in human proximal tubule cells possibly due to a decreased expression of TGF-βRI. In addition, BMP-7 was able to reverse TGF-β-induced phosphorylation of Smad 2.

**Conclusions:**

The findings suggest a protective role for BMP-7 by counteracting the TGF-β and TNF-α-induced negative effects. The reduced expression of BMP-7 in patients with hypertensive nephrosclerosis may imply loss of protection and regenerative potential necessary to counter the disease.

## Background

Bone Morphogenetic Protein (BMP)-7 has been found to be renoprotective and to promote kidney regeneration in several animal models [1]. This finding was also observed in acute renal injury of the adult kidney as well as in chronic kidney disease [2-4]. The following mechanisms have been found to play a role in the effect of BMP-7: 1) regeneration of tubular epithelial cells by reversal of the epithelial-to-mesenchymal transition (EMT) [4], 2) decrease of apoptosis in tubular epithelial cells [5] and 3) anti-inflammatory effects by decreasing the accumulation of inflammatory cells [3,6] and an amelioration of TNF-α-induced expression of pro-inflammatory cytokines in proximal tubular cells [7].

However, these results have usually been obtained in animal models, and data from human kidneys are scarce. The available experiments in human tissue have shown a tubular expression pattern of BMP-7 in normal kidneys [8] and a reversibility of TGF-β-induced EMT by BMP-7 in proximal tubular cells [9], which was similar to the results obtained in rodents. On the other hand, in contrast with some prior animal data, some research has shown an increased expression of BMP-7 in proximal tubular cells in patients with proteinuria [10] and a failure to attenuate a TGF-β-induced EMT in primary or immortalised human proximal tubule epithelial cells *in vitro *[11].

The aim of the present study was to comprehensively investigate BMP-7 expression as well as its regulation and function in normal and hypertensive sclerotic human kidneys [12,13].

## Methods

### Patients and Tissues

Bioptic kidney samples for immunostaining were obtained from 12 patients with clinically confirmed nephrosclerosis. Control tissue was obtained from patients undergoing surgical nephrectomy for neoplastic kidney disease (n = 10).

Kidney tissue for real-time PCR was obtained from the European Renal cDNA Bank [14]. We included 32 samples from patients with nephrosclerosis and 10 samples from pretransplant biopsies of living and deceased donors. None of the donors had relevant proteinuria, decreased renal function or arterial hypertension [14]. The characteristics of patients with nephrosclerosis are displayed in Table 1.

**Table 1:** Characteristics of patients with nephrosclerosis

	Real time PCR (European Renal cDNA Bank)	Immunohisto-chemistry
Number of patients	32	12
Age in years (median, IQR)	55 (48-64)	64.5 (58.5-67.5)
Male/female (n)	25/7	9/3
Disease duration (years)	n.a.	14 (7-25.8)
Creatinine Clearance (ml/min) (median, IQR)	51.8 (32.8-61.8)	39.0 (15.5-56.4)
Proteinuria (g/day) (median, IQR)	2.3 (0.4-3.1)	0.5 (0.2-1.4)
Degree of interstitial fibrosis (%) (median, IQR)	n.a.	43.6 (39.8.-48.5)

*Legend: *IQR, interquartile range; n.a., not available

Reference values: Creatinine Clearance: 80-140 ml/min.,

Proteinuria: <150 mg/day

The use of human samples was approved by the ethical committee of the Georg August University Göttingen (Ref-No #11/10/04).

### Cell culture experiments

The human proximal tubular epithelial cell line HK-2 was cultured in serum free complete Quantum 286 medium (PAA, Pasching, Germany) [15]. Cells were made quiescent 24 hours prior to stimulation by incubation with DMEM medium without additives (Invitrogen, Carlsbad, USA).

### Immunofluorescence

Double immunofluorescence (DIF) with anti BMP-7 (goat polyclonal IgG, Santa Cruz, USA) was performed to identify the expression localisation of BMP-7 inside the normal and nephrosclerotic kidney. Counterstaining with Aquaporin-1 (AQ-1, diluted 1:50; rabbit polyclonal IgG, Alpha Diagnostic, San Antonio, USA) was used to identify the proximal and distal tubule, and counterstaining with Aquaporin-2 (AQ-2, diluted 1:50, rabbit polyclonal IgG, Calbiochem, San Diego, USA) was used to identify the collecting duct [16]. Double staining was performed using a combination of rhodamine red for BMP-7, and FITC (green) for AQ-1 and -2. Double-labelled cells resulted in orange staining. Negative controls were included in all experiments by exclusive incubation with the second antibody.

To analyse EMT, HK-2 cells (10,000/ml) were stained for the epithelial marker zona occludens-1 (ZO-1, 1:25, rabbit polyclonal IgG, Santa Cruz, Santa Cruz, USA) and the mesenchymal marker α-smooth muscle actin (α-sm actin, 1:250, rabbit monoclonal IgG, Novus Biologicals, Littleton, USA) after stimulation for 48 hours with TGF-β (10 ng/ml) in the presence or absence of BMP-7 (10, 100 ng/ml).

To analyse the influence of BMP-7 on cell death, HK-2 cells (10,000/ml) were stained for annexin-V (Annexin-V-FLUOS Staining Kit, Roche, Mannheim, Germany) after stimulation with TNF-α (20 ng/ml) for 24 hours in the presence or absence of BMP-7 (1, 10, 100 ng/ml, R&D, Minneapolis, USA). Staining for TGF-β receptor type I (TGF-βRI, 1:50, rabbit polyclonal IgG, Santa Cruz, Santa Cruz, USA) was performed after incubation with BMP-7 (100 ng/ml) for 48 hours.

In both kidney slices and HK-2 cells, cell nuclei (blue) were identified by counterstaining with 4,6-diamino-2-phenylindolyl-dihydrochloride (DAPI; Vector Labs, Burlingame, USA). Staining was visualised by epifluorescence microscopy (Zeiss Axiovert S100TV, Oberkochen, Germany). Quantification of cell staining intensity was given in grey values by the software AnalySIS (Olympus Soft Imaging Solutions GmbH, Münster, Germany). For comparison between the single stimulation experiments, the same number of cells was evaluated.

### Immunohistochemistry

To identify differences in the BMP-7 expression pattern of sclerotic and normal kidneys, immunohistochemistry (IMH) was performed. The IMH of BMP-7 was performed as described [16]. An antibody against human BMP-7 (see DIF) was used as the primary antibody, and rabbit anti-goat biotinylated Ig antibody (DAKO, Glostrup, Denmark) was used as the secondary antibody. Negative controls were included in all experiments, and for these, the first antibody was omitted. Nuclear counterstaining was performed using hematoxylin. Intensity of BMP-7 expression in tubules were evaluated by an established semiquantitative score as follows: 0 = no staining; 1 = weak staining; 2 = moderate staining and 3 = strong staining [17]. For evaluation, five visual field areas per kidney slice were analysed, and the results are presented as the mean ± standard error.

### Real-time PCR

Real-time RT-PCR was performed on a TaqMan ABI 7700 Sequence Detection System (PE Biosystems, Weiterstadt, Germany) using heat-activated TaqDNA polymerase (Amplitaq Gold; PE Biosystems). After an initial hold of two minutes at 50°C and ten minutes at 95°C, the samples were cycled 40 times at 95°C for 15 seconds and 60°C for 60 seconds. Quantification was performed using the standard curve approach applying serial dilutions of standard cDNA for each target and reference gene. All samples, controls and patients, were analysed in the same run in technical replicates with high reproducibility (threshold cycle (Ct) difference <0.4; mean Ct of each sample <32) [14,16]. To exclude the possibility of genomic contamination, we conducted a "no-RT control" in every single PCR using non-reverse transcribed mRNA instead of cDNA. Only real-time PCRs with a negative "no-RT control" were evaluated.

Quantification of BMP-7 mRNA transcription in kidney tissue samples was determined separately for glomeruli and for the tubulointerstitium after manual microdissection [14]. Thereby, tubulointerstitial and glomerular samples were from the same individuals. Quantification of BMP-7 transcription in HK-2 cells by real-time PCR was done after stimulation with angiotensin-II (AT-II, 10^-7^, 10^-5 ^and 10^-3 ^M, Sigma Aldrich, St. Louis, USA), with the angiotensin-II receptor antagonist telmisartan (10, 20 and 30 μM, Boehringer Ingelheim, Germany) or both in combination (10^-3 ^M AT-II + 10, 20 and 30 μM telmisartan) for 12 hours [14,16].

To evaluate EMT in human HK-2 cells, quantification of the epithelial marker ZO-1, and E-cadherin as well as the mesenchymal marker α-sm actin and S100A4 was done after stimulation with TGF-β (10 ng/ml), BMP-7 (100 ng/ml) or both in combination (10 ng/ml TGF-β + 100 ng/ml BMP-7). Moreover, TGF-βRI was determined after stimulation with 1, 10 and 100 ng/ml BMP-7 in HK-2 cells. Total RNA from both tissue and cells was extracted using the Qiagen RNeasy Mini Kit, including a treatment with RNase-Free DNase (both Qiagen, Hilden, Germany) and reverse transcribed using random priming. Oligonucleotides were obtained from Primerdesign (Southampton, UK) and are listed in Table 2. The housekeeping gene Peptidyl-Prolyl-Isomerase-A (PPIA) was chosen because its expression level was comparable to BMP-7 and validated in kidney tissue with a constant expression [18].

**Table 2:** Oligonucleotides

mRNA	Gene Bank accession number	Oligonucleotide (5' - 3') (up/down)								bp
PPIA	NM 021130	TGG	GCA	ACA	TAG	TGA	GAC	G		139
		TGT	ACA	GTG	GCA	TGA	TAA	TAG	C	
BMP-7	NM 001719	CCT	CCA	TTG	CTC	GCC	TTG			114
		TAT	GCT	GCT	CAT	GTT	TCC	TAA	TAC	
E-cadherin	NM 00436	CAT	GAG	TGT	CCC	CCG	GTA	TC		89
		CAG	TAT	CAG	CCG	CTT	TCA	GA		
ZO-1	NM 003257	AAA	CAA	GCC	AGC	AGA	GAC	C		95
		CGC	AGA	CGA	TGT	TCA	TAG	TTT	C	
α-sm-actin	NM 001613	AAG	CAC	AGA	GCA	AAA	GAG	GAA	T	76
		ATG	TCG	TCC	CAG	TTG	GTG	AT		
S100A4	NM 002961	TCT	TTC	TTG	GTT	TGA	TCC	TGA	CT	130
		AGT	TCT	GAC	TTG	TTG	AGC	TTG	A	
TGF-βRI	NM 004612	TGA	CTG	AAG	GCT	GCT	CTG	G		125
		CAT	CTG	CTC	AAT	CTC	CAA	ACT	TG	

### Protein extraction and immunoblotting

Western blot analysis of phosphorylated Smad 1/5/8 (pSmad 1/5/8, diluted 1:1000; rabbit polyclonal IgG, Cell Signalling, Beverly, USA) and phosphorylated Smad 2 (pSmad 2, diluted 1:1000; rabbit polyclonal IgG, Cell Signaling, Beverly, USA) was performed as previously described [17]. Polyclonal goat anti-rabbit IgG antibody (1:2000, DAKO, Glostrup, Denmark) was used as secondary antibody. Phosphorylated Smad 1/5/8 was determined in HK-2 cells (1,000,000/ml, 25 μg/blot) after stimulation with AT-II (10^-3 ^M) for 36 hours. Phosphorylated Smad 2 was determined in HK-2 cells (1,000,000/ml, 25 μg/blot) after stimulation with 10 ng/ml TGF-β alone and in combination with BMP-7 (100 ng/ml).

### FACS analysis of apoptosis

FACS analysis was performed to determine the rate of apoptosis in HK-2 cells [19]. Cells were stimulated with either TNF-α (20 ng/ml, R&D, Minneapolis, USA), BMP-7 (1, 10, 100 ng/ml), or both for 24 hours. Apoptotic cells were classified as annexin-V positive and propidium iodide negative using a FACScan flow cytometer (Becton Dickinson, San Jose, CA).

### Statistical analysis

Statistical analysis was performed using SigmaStat (Systat Software Inc., San Jose, USA). Real-time PCR data of BMP-7 expression in the whole kidney were evaluated using the Kruskal-Wallis test followed by the Mann-Whitney-U test for comparison. These data and the clinical data were presented in median with quartiles (25^th ^and 75^th ^percentile). The results of the cell culture experiments were presented as the means with standard errors. Significant changes were evaluated using Student's t-test due to the sample sizes (minimum: n = 3) and can only be regarded as descriptive. For both statistical methods, p-levels of < 0.05 were regarded to be significant.

## Results

### BMP-7 mRNA level is decreased in nephrosclerotic compared to normal kidneys

**Figure 1:**
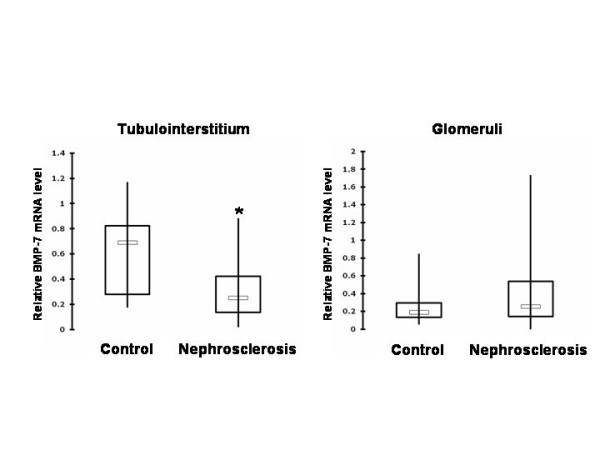
**Real-time PCR analysis: the BMP-7 mRNA level is decreased in the tubulointerstitium but not in the glomeruli of patients with sclerotic compared to normal kidney tissue**. Real time PCR with BMP-7 in normal kidneys (n = 10) and those from patients with nephrosclerosis (n = 32), housekeeping gene PPIA. Relative BMP-7 mRNA transcription in the tubulointerstitium and glomeruli of the kidneys are shown. BMP-7 mRNA was significantly decreased in the tubulointerstitium of patients with nephrosclerosis compared to normal kidneys (p < 0.01; Kruskal-Wallis test followed by Mann-Whitney-U test). The BMP-7 mRNA level in the glomeruli did not differ significantly. The results are displayed as medians with quartiles.

BMP-7 mRNA was significantly reduced in the tubulointerstitium of patients with nephrosclerosis (relative median quantity with quartiles: 0.25 (0.14 - 0.42)) compared to normal kidneys (0.69 (0.28 - 0.83), p < 0.01) (Figure 1).

Levels of glomerular BMP-7 mRNA did not significantly differ among patients with nephrosclerosis (0.26 (0.14 - 0.54)) compared to those without kidney diseases (0.19 (0.13 - 0.30), p = 0.561) (Figure 1).

### BMP-7 is mainly expressed in the distal tubule and collecting duct

**Figure 2:**
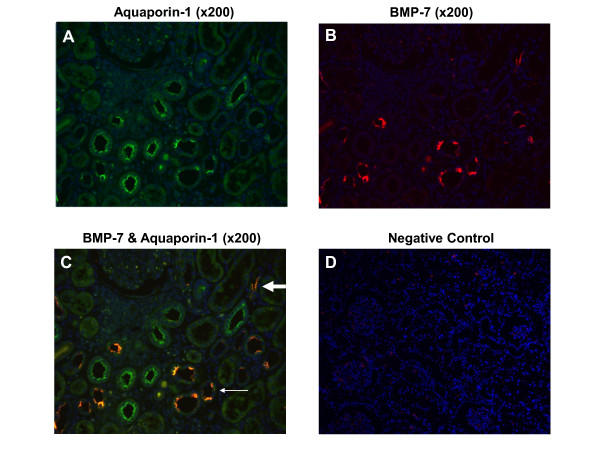
**Immunofluorescence of BMP-7 and Aquaporin 1 in normal kidneys revealing expression in distal tubules and proximal tubular cells.** Double immunofluorescence of BMP-7 with aquaporin-1 (AQ-1): **A**) AQ-1 is green (FITC), **B**) BMP-7 staining is red (rhodamine red), **C**) double labelling of BMP-7 and AQ-1 is orange and nuclei is blue (DAPI). Parallel binding of BMP-7 and AQ-1 is seen mainly in tubules with smaller cuboidal cells and larger lumen indicating expression in distal tubules (small arrow) and less in proximal tubule cells (bulky arrow). Staining was low in the glomeruli. Original magnification: × 200. **D**) Negative control × 100.

**Figure 3:**
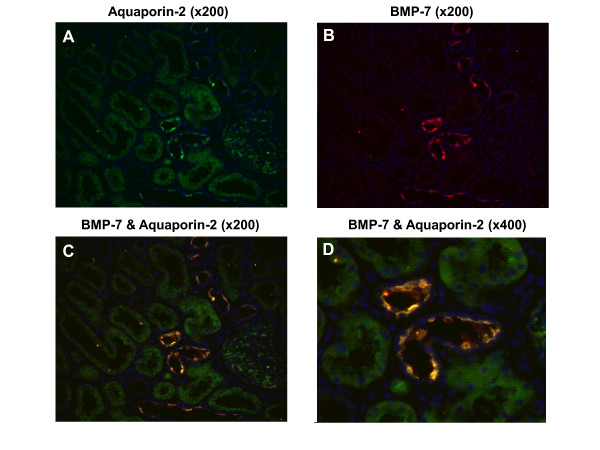
**Immunofluorescence of BMP-7 and Aquaporin 2 in normal kidneys showing expression in the collecting duct**. Double immunofluorescence of BMP-7 with aquaporin-2 (AQ-2): **A)** AQ-2 is green (FITC), **B)** BMP-7 staining is red (rhodamine red), C, D) double-labelling of BMP-7 and AQ-2 is stained orange (**C**: × 200, **D**: × 400) and nuclei are blue (DAPI). Parallel binding of BMP-7 and AQ-2 revealing expression of BMP-7 in the collecting duct. Negative control: see Figure 2D. Original magnification: × 200 and × 400.

**Figure 4:**
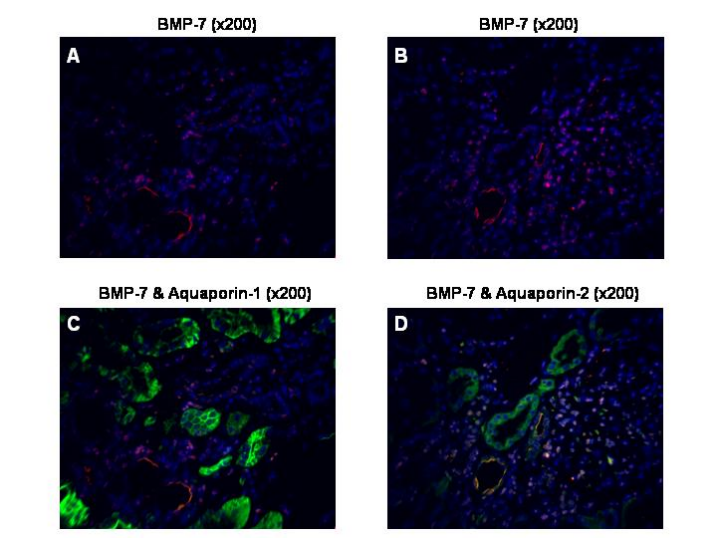
**Immunofluorescence of BMP-7 and Aquaporin 1 and Aquaporin 2 in nephrosclerotic kidneys showing decreased expression of BMP-7**. Double immunofluorescence of BMP-7 with aquaporin-1 (AQ-1) and aquaporin-2 (AQ-2): BMP-7 staining is red (rhodamine red, **A, B**), double-labeling of BMP-7 and AQ-1 (**C**) or AQ-2 (**D**) is stained organge. Expression of BMP-7 was lower than in the control kidneys demonstrated by the lower staining intensity of BMP-7 despite a longer exposure time and therewith a stronger intensity of AQ-1 and AQ-2. Original magnification: × 200.

**Figure 5:**
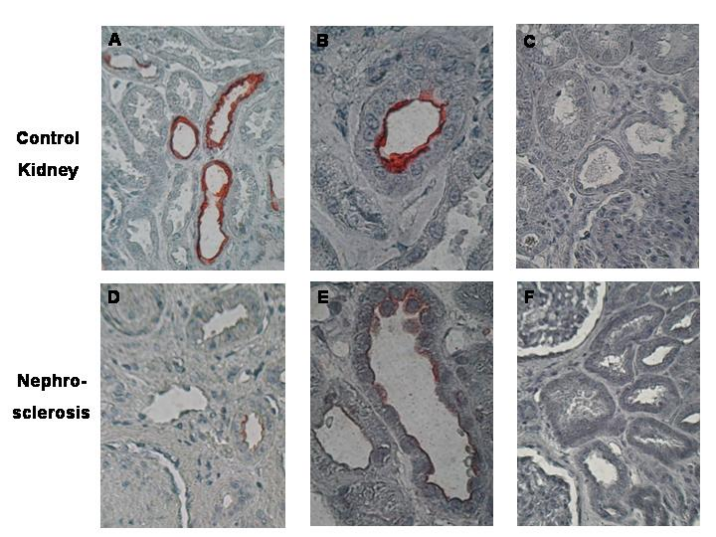
**Immunohistochemistry of BMP-7 with unchanged localisation, but lower expression levels in patient with nephrosclerosis compared to normal kidneys**. Immunostaining for BMP-7 was done in 10 normal kidneys **(A - C) **and in 12 kidneys from patients with nephrosclerosis **(D - F)**. Expression levels of BMP-7 were low in all examined kidney slices. BMP-7 is located mainly endoluminally in the normal kidney (**A**: × 400; **B**: × 1000). In kidneys of patients with nephrosclerosis, localisation of BMP-7 expression was unchanged, but lower than in the control kidneys (**D**: × 400; **E**: × 1000). Negative controls did not show any staining (**C**, **F**).

In normal kidneys, BMP-7 protein was found to be localised in the epithelium of the distal tubule and the collecting duct but was less abundant in the proximal tubular cells or glomeruli. Expression in the distal tubules was suggested by parallel binding of BMP-7 and AQ-1 in tubules with smaller cuboidal cells and larger lumen. Proximal tubules with prominent cuboidal epithelial cell lining and the smaller lumen showed higher AQ-1 than BMP-7 expression (Figure 2). Double immunofluorescence (DIF) with BMP-7 and AQ-2 showed parallel expression, revealing expression of BMP-7 in the collecting duct (Figure 3). As illustrated by double immunofluorescence (Figure 2, 3, 4) and immunohistochemistry (Figure 5), localisation of the BMP-7 expression was unchanged in patients with nephrosclerosis, but the mean intensity was significantly lower (0.86 ± 0.17) compared to controls (2.5 ± 0.07) (95% confidence interval (CI) 0.23 - 3.10, p < 0.05).

### Regulation of BMP-7 expression by the renin-angiotensin system

**Figure 6:**
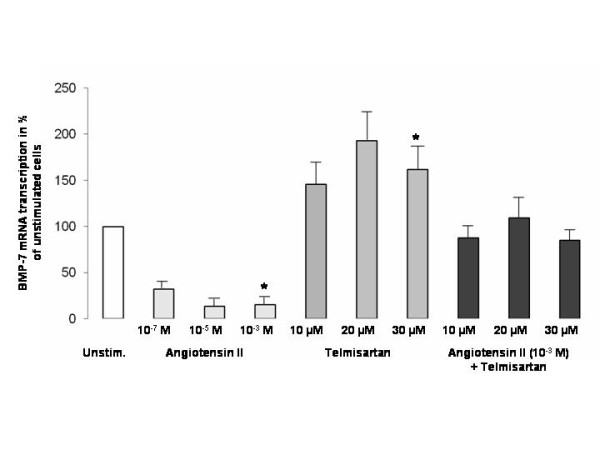
**BMP-7 mRNA level in HK-cells is decreased after stimulation with angiotensin-II and increased after stimulation with telmisartan**. BMP-7 mRNA level in the proximal tubule cell line HK-2 was determined by real-time PCR (housekeeping gene PPIA) after stimulation with angiotensin-II (10^-7^, 10^-5^, 10^-3 ^M) and telmisartan (10, 20, 30 μM) or with both combined for 12 hours (n = 12). BMP-7 mRNA level is significantly decreased after stimulation with 10^-3 ^M AT-II (p < 0.001) and increased after stimulation with 30 μM telmisartan (p < 0.01). Co-stimulation of AT-II and telmisartan showed non-significantly increased BMP-7 mRNA levels compared to stimulation with AT-II alone. The results are presented as the mean % of unstimulated cells ± standard error.

To examine the interaction of BMP-7 with the renin-angiotensin system, BMP-7 mRNA level in HK-2 cells was quantified by real-time PCR (18 μg/ml cDNA; 25 - 30 cycles) after stimulation with AT-II, telmisartan or both in combination for 12 hours. As shown in Figure 6, the transcription of BMP-7 was significantly decreased after stimulation with 10^-3 ^M AT-II compared to unstimulated cells (95% confidence interval (CI) 66.1 - 103.3, p < 0.001) and increased after stimulation with 30 μM telmisartan (95% CI 29.6 - 146.2, p < 0.01). The stimulation with a combination of AT-II and telmisartan showed higher BMP-7 mRNA levels compared to AT-II alone; the difference was, however, not significant.

### BMP-7 is able to reverse EMT in human proximal tubule cells (HK-2)

**Figure 7:**
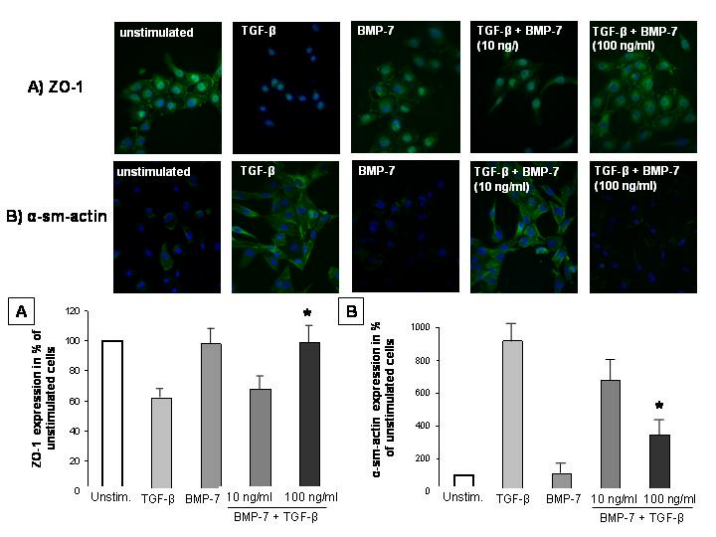
**BMP-7 reverses TGF-β-induced EMT detected by immunofluorescence**. BMP-7 reverses TGF-β-induced EMT as demonstrated by immunofluorescence of ZO-1 (**A**) and α-sm actin (**B**) in human proximal tubular cells (HK-2, n = 3). Stimulation with TGF-β alone (10 ng/ml) led to a distinctive morphological transformation into fibroblastoid cells in combination with a lowered expression of ZO-1 (**A**) and a higher expression of α-sm actin (**B**). Reversal of EMT was demonstrated by co-stimulation of HK-2 cells with TGF-β (10 ng/ml) and BMP-7 (10, 100 ng/ml) showing epithelial morphology. Co-Stimulation with 10 ng/ml TGF-β and 100 ng/ml BMP-7 were leading to a significantly higher expression of ZO-1 (p < 0.01; **A**) and lower expression of α-sm-actin (p < 0.001; **B**) compared to TGF-β stimulation alone. BMP-7 alone did not substantially influence the expression of ZO-1 and α-sm-actin. Values were evaluated by determination of the staining intensity per cell using Student's t-test and are presented as % of unstimulated cells.

**Figure 8:**
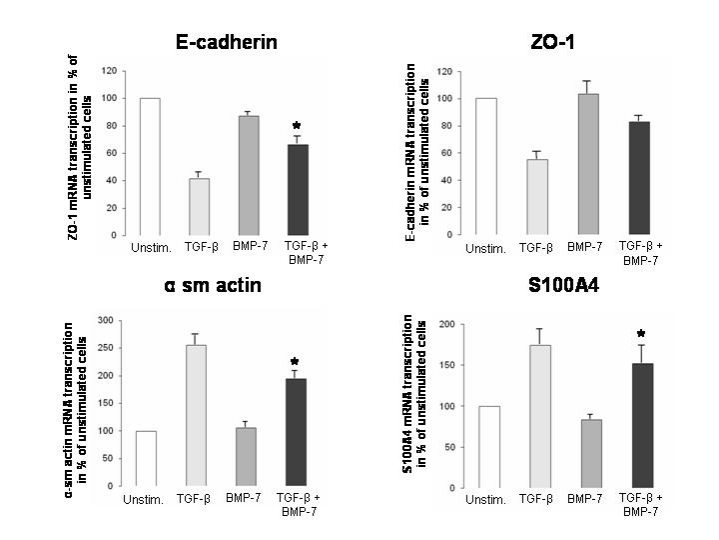
**Real time PCR indicating reversal of TGF-β induced EMT by BMP-7**. The mRNA level of the epithelial marker ZO-1 and E-cadherin and the mesenchymal marker α-sm-actin and S100A4 were analysed after stimulation of HK-2 cells with TGF-β alone, BMP-7 alone or both in combination (n = 3). There was a decrease of ZO-1 and E-cadherin and an increase of α-sm-actin and S100A4 mRNA after stimulation with TGF-β alone, while no significant effect was seen after stimulation with BMP-7 alone. Co-stimulation of TGF-β and BMP-7 led to a significant increase of ZO-1 (p < 0.001) and E-cadherin (p = 0.05) as well as a decrease of α-sm-actin (p < 0.001) and S100A4 mRNA (p < 0.05) compared to TGF-β alone. The results were analysed by Student's t-tests and are displayed as % of unstimulated cells.

To analyse the influence of BMP-7 on EMT in human proximal tubule cells, the expression of epithelial and mesenchymal marker were determined after stimulation with BMP-7 alone or in combination with TGF-β. Stimulation of human proximal tubule cells (HK-2) with TGF-β (10 ng/ml) alone induced EMT as demonstrated by morphological transformations of epithelial into fibroblastoid cells (Figure 7). This morphological observation is underscored by the lower mRNA level of the epithelial markers ZO-1 and E-cadherin and higher transcription of the mesenchymal markers α-sm-actin and S100A4 (Figure 8).

Co-stimulation of TGF-β and BMP-7 led to a reversal of the prior morphological transformation with a predominant epithelial phenotype (Figure 7). The reversal of EMT was further demonstrated by significantly increased mRNA levels of E-cadherin (95% CI: 32.2 - 17.6, p < 0.001), by a tendency for increased mRNA levels of ZO-1 (95% CI: 0.5 - 56.5, p = 0.05) and by significantly decreased levels of α-sm-actin (95% CI: 28.1 - 93.8, p < 0.001) and S100A4 (95% CI: 9.6 - 34.7, p < 0.05). In analogy, similar changes could be detected for the protein level of ZO-1 (95% CI: 5.7 - 69.1, p < 0.01) and α-sm-actin (95% CI: 284.7 - 859.6, p < 0.05) (Figure 8). Both the expression of ZO-1 and α-sm-actin showed dose-dependent effects. Significant changes in both protein and mRNA level were observed only after stimulation with a concentration of 100 ng/ml BMP-7. BMP-7 alone did not substantially influence the mRNA level of ZO-1, E-cadherin, α-sm-actin and S100A4 (Figure 8), nor did it effect the protein level of ZO-1 and of α-sm-actin compared to unstimulated cells (= 100%) (Figure 7).

### Decreased expression of pSmad 1/5/8 after stimulation with BMP-7

**Figure 9:**
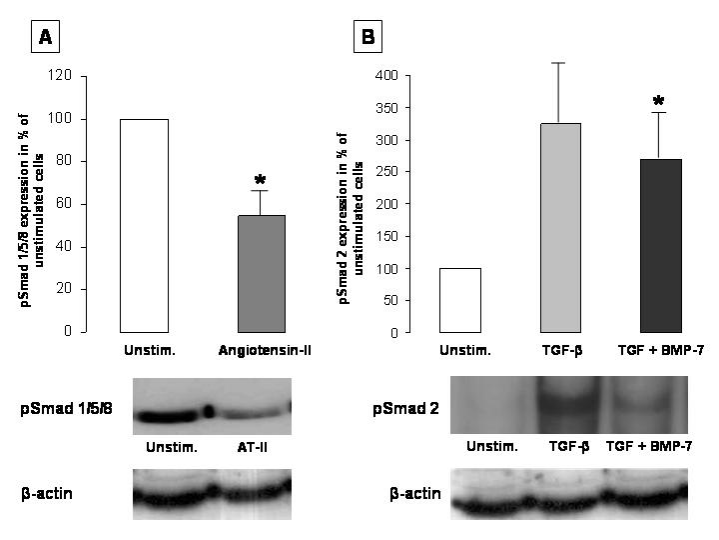
**Figure 9A - Smad 1/5/8 is decreased by angiotensin-II**. Western Blotting was performed in HK-2 cells for phosphorylated Smad 1/5/8 after stimulation with AT-II (10^-3^ mM, n = 3). Phosphorylated Smad 1/5/8 was significantly decreased in HK-2 cells after stimulation with AT-II (p < 0.05). Figure 9B - BMP-7 attenuates TGF-β induced pSmad 2. Western blotting was performed in HK-2 cells for phosphorylated Smad 2 after stimulation with TGF-β alone (10 ng/ml) and TGF-β in combination with BMP-7 (100 ng/ml, n = 3). Phosphorylated Smad 2 was increased after stimulation with TGF-β alone and significantly decreased after stimulation with TGF-β in combination with BMP-7 (p < 0.05).

To further verify the decrease of BMP-7 by angiotensin-II, we examined the expression of phosphorylated Smad 1/5/8 as intracellular pathway mediators of BMP-7 by western blotting (n = 3). In concordance with the decreased BMP-7 expression by AT-II, phosphorylated Smad 1/5/8 was significantly decreased in HK-2 cells after stimulation with 10^-3 ^mM AT-II to 54.8 ± 9.8% of the unstimulated controls (= 100%, 95% CI: 21.0 - 70.2, p < 0.05) (Figure 9A).

### BMP-7 decreases TGF-β Receptor type I in HK-2 cells

**Figure 10:**
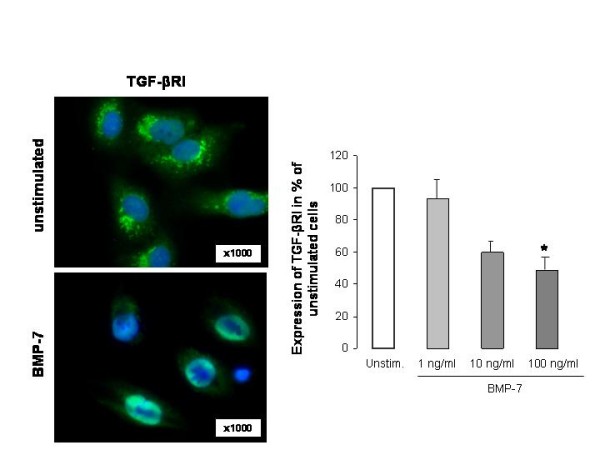
**BMP-7 decreases expression of TGF-β receptor type I**. Expression of TGF-β receptor type I after stimulation with BMP-7 (1, 10, 100 ng/ml) detected by double immunofluorescence: quantification of cell staining intensity was given in grey values by the software AnalySIS. Expression rate is changed compared to unstimulated cells, concomitant with reduced staining intensity (100 ng/ml: p < 0.01). Original magnification: × 1000. Results were analysed by Student's t-test and are displayed as the % of unstimulated cells in the mean ± standard error.

To test the hypothesis that reversal of EMT by BMP-7 may be mediated by the down-regulation of TGF-β Receptor type I (TGF-βRI) expression, this expression was analysed after stimulation of HK-2 cells with BMP-7. Expression of TGF-βRI was decreased after stimulation with BMP-7 in a dose-dependent manner compared to unstimulated cells (= 100%). This was demonstrated at the protein level by immunofluorescence (Figure 10) and at the mRNA level by real-time PCR (1 ng/ml: 92.98 ± 21.60%, 10 ng/ml: 39.29 ± 2.60%, 100 ng/ml: 25.71 ± 8.37%, data not shown). Thereby, stimulation with 100 ng/ml leads to a significant decrease of TGF-βRI expression at both the protein (95% CI: 27.3 - 75.6, p < 0.01) and the mRNA level (95% CI: 53.5 - 95.1, p < 0.01) compared to unstimulated HK-2 cells (Figure 10). Moreover, the expression pattern of TGF-βRI changed after stimulation with BMP-7 to a localisation that was more intracellular than on the cell surface.

### Decreased pSmad-2 expression in HK-2 cells after stimulation with BMP-7

To further test the hypothesis that the decreased expression of the TGF-β receptor type I by BMP-7 may have functional consequences, phosphorylated Smad 2 was determined by western blotting after stimulation with TGF-β in presence or absence of BMP-7. Compared to unstimulated HK-2 cells, phosphorylated Smad 2 was increased after stimulation with TGF-β alone (323.9 ± 96.4%) and significantly decreased after co-stimulation with TGF-β and BMP-7 (95% CI: 34.8 - 52.8, p < 0.05; Figure 9B).

### BMP-7 influence on TNF-α induced apoptosis

**Figure 11:**
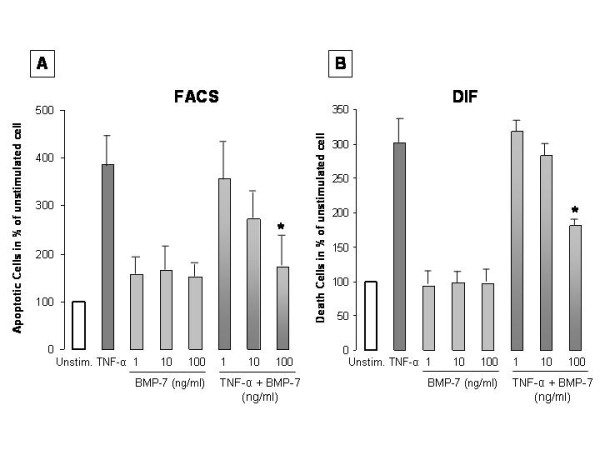
**BMP-7 deceases TNF-α induced apoptosis**. Induction of apoptosis (A) and cell death (B) in proximal tubular cells (HK-2) after stimulation with 20 ng/ml TNF-α alone and/or in combination with BMP-7 (1, 10, 100 ng/ml). Apoptotic cells were detected by FACS analysis counting annexin-V positive and propidium iodide negative cells (n = 3). Cell death was detected after staining with annexin-V (n = 3). TNF-α alone induced high rates of apoptosis and cell death. Co-stimulation with TNF-α and BMP-7 resulted in significant dose-dependent lower rates of apoptosis and cell death compared to TNF-α alone (DIF: p < 0.05, FACS: p < 0.05). The results were analysed by Student's t-test and are displayed as the % of unstimulated cells.

Since BMP-7 is known to have protective effects on tubular epithelial cells, we examined the influence of BMP-7 on cell death and apoptosis in proximal tubule cells (HK-2) after stimulation with TNF-α alone and in combination with BMP-7. Apoptotic cells were detected by FACScan (FACS) counting annexin-V positive and propidium iodide negative cells (Figure 11A): TNF-α (20 ng/ml): 386.6 ± 41.3%; TNF-α (20 ng/ml) + 1 ng/ml BMP-7: 356.1 ± 53.3%; + 10 ng/ml: 272.0 ± 28.9%; + 100 ng/ml: 173.2 ± 48.4%, 95% CI: 57.0 - 369.8%, p < 0.05), each compared to unstimulated cells.

The influence of BMP-7 on TNF-α induced cell death of HK-2 cells was determined by immunofluorescence after staining for annexin-V (Figure 11B). Stimulation of the proximal tubule cells with 20 ng/ml TNF-α led to cell death rates of 302.2 ± 1.5% compared to unstimulated controls. Co-stimulation with a combination of TNF-α (20 ng/ml) and BMP-7 (100 ng/ml) resulted in significantly lower cell death rates than those observed with TNF-α stimulation alone (95% CI: 52.7 - 189.3%, p < 0.05). Thus, the effect was dose-dependent: TNF-α + 1 ng/ml BMP-7: 318.0 ± 2.1%; + 10 ng/ml BMP-7: 283.4 ± 6.4%; +100 ng/ml BMP-7: 181.2 ± 0.7%).

BMP-7 alone did not substantially induce cell death (DIF, 100 ng/ml: 97.5 ± 3.9%) or apoptosis (FACS, 100 ng/ml: 152.34 ± 5.9%).

## Discussion

In this study, expression, regulation and function of BMP-7 was compared in human hypertensive nephrosclerotic versus normal kidneys. Our results demonstrated the following: 1) renal BMP-7 is decreased in human hypertensive nephrosclerosis; 2) BMP-7 transcription is regulated by the renin-angiotensin system (AT-II vs. telmisartan) *in vitro*; 3) BMP-7 is able to reverse TNF-α- and TGF-β-induced effects in human proximal tubule cells, albeit only high concentrations; and 4) BMP-7 decreased TGF-βRI expression.

### BMP-7 localisation in normal kidneys and nephrosclerosis

BMP-7 was found to be expressed predominantly in the epithelium of the distal tubule, the collecting duct and less in the proximal tubular cells in normal kidneys. This expression pattern is concordant with recently published data, demonstrating the same expression pattern in normal human kidneys [8]. We found that localisation of BMP-7 expression was unchanged in human hypertensive nephrosclerosis, but expression was reduced, as demonstrated by immunostaining and real-time PCR. Our results are in accordance with findings in animals, where the renal BMP-7 expression was reduced in several kidney disease models, including acute ischemic renal injury, tubulointerstitial fibrosis and diabetic nephropathy [2,20,21]. In contrast, previous investigations by Rudnicki et al. in proteinuric patients showed an increase of BMP-7 expression in proximal tubule cells [10]. Due to the heterogeneity of the possible underlying kidney disease [10] in the different studies, it is difficult to assess and compare these findings. Moreover, a reason for the discrepancy of the results could be disparities in the stage of examined kidney disease. Thus, progression of kidney fibrosis is associated with a concomitant loss of BMP-7 expression in later stages of kidney disease [22]. Experiments with streptozotocin-induced diabetes in rodents demonstrated decreased renal expression of BMP-7 to 50% of its original level at week 15, and up to 10% of the control animals by week 30 [22]. This, in turn, could be due to the TGF-β induced down-regulation of BMP-7 as demonstrated in proximal tubule cells [22].

### BMP-7 and the role of the renin-angiotensin system

To further test our hypothesis of BMP-7 involvement in the development of human hypertensive nephrosclerosis, we performed tissue culture experiments. In preliminary experiments, we were able to detect BMP-7 mRNA in primary isolated human distal tubule cells [23], but the number of cells were limited; moreover, we were not able to cultivate these cells long enough to perform stimulation experiments. Due to the unavailability of human distal tubule cell lines, we used the human proximal cell line HK-2, although results on BMP-7 mRNA transcription in proximal tubular epithelial cells are controversial. Whereas Wetzel et al. failed to show any BMP-7 mRNA in primary and immortalised (HK-2) proximal tubule cells [8], BMP-7 mRNA was detected by Rudnicki et al. [10]. In our experiments, BMP-7 transcription in HK-2 cells was present, but copy number was found to be low. Despite the low mRNA level, BMP-7 was significantly decreased after stimulation with AT-II, which could explain the lower BMP-7 expression in the kidneys of patients with hypertensive nephrosclerosis. The effect may be evident from the decreased expression of the pSmads 1/5/8 after stimulation with angiotensin-II. However, the Smad complex 1/5/8 is also activated by pro-fibrotic BMPs (e.g., BMP-2, -4) and the exact regulation is due to several confounding factor [24,25].

Increased BMP-7 transcription after co-stimulation with AT-II and the angiotensin-II receptor antagonist telmisartan may, in part, explain the protective effects of these drugs in kidneys. However, significantly increased BMP-7 mRNA level was seen after stimulation with telmisartan alone, revealing an AT-II independent effect of telmisartan on BMP-7 expression. These findings are in line with former studies, although most authors have focused primarily on the telmisartan-induced decrease of inflammatory cytokines and less on protective growth factors. Hence, AT-II independent effects of telmisartan were described to decrease TNF-α induced expression of interleukin-6 in vascular smooth muscle cells [26], as well as the expression of monocyte chemoattractant protein 1 (MCP-1, CCL-2) and its receptor CCR2 in peripheral blood monocytes [27].

### BMP-7 effect on EMT

Thus far, the data on the reversibility of EMT by BMP-7 in human kidneys are controversial. Although research by Xu [9] and Veerasamy [28] showed that BMP-7 attenuated TGF-β-induced EMT in human proximal tubule cells (HK-2), Dudas was not able to confirm these results in primary and immortalised human proximal tubule cells [11]. Our functional data showed that BMP-7 was able to reverse the TGF-β-induced EMT in human proximal tubule cells by using high concentrations of recombinant BMP-7 (100 ng/ml). One possible mode of action can be the decreased expression of the TGF-β receptor type I, which may also contribute to the reversal of EMT by BMP-7. Thereby, the attenuated TGF-β signalling may be caused by the decreased expression level as well as by the decreased receptor expression on the cell surface. This hypothesis may be in line with previous findings suggesting that TGF-β R1 is internalised in clathrin-coated vesicles and that the process of being expressed on the cell surface plays an important regulatory role in TGF-β signalling [29]. The functional aspect may be demonstrated by the decreased expression of phosphorylated Smad 2 after stimulation with TGF-β and BMP-7 compared to TGF-β alone.

In contrast to Dudas [11], Xu et al. and our working group used concentrations as high as 100 ng/ml BMP-7 to attenuate TGF-β-induced EMT in HK-2 cells [9]. This high dose of BMP-7 may have inhibited the systemic administration as a therapeutic option for chronic kidney disease. However, of course, in vitro studies in cell lines are not necessarily transferable to in vivo studies in organisms.

### BMP-7 effect on apoptosis

In addition, data from our study revealed that BMP-7 counteracts TNF-α-induced apoptosis in human proximal tubule cells. These results in human cells are in accordance with those reported by Vukicevic and co-workers, who demonstrated decreases in apoptotic cells in BMP-7 treated subjects in rodents with acute renal failure [3]. However, again, high concentrations of BMP-7 were necessary to achieve significant changes.

## Conclusions

Our findings suggest a protective role of BMP-7 by counteracting TGF-β and TNF-α negative effects *in vitro*, although high concentrations of BMP-7 are necessary. In agreement with the findings in animal studies, the reduced expression of BMP-7 in patients with hypertensive nephrosclerosis may imply a loss of protection and regenerative potential necessary to counter the disease. The decrease may be in part induced by angiotensin-II and attenuated by telmisartan.

## Competing interests

The authors declare that they have no competing interests.

## Authors' contributions

CPB and GAM outlined the study design; MK, MK, KL, HS and CDC gave important input into the study design; CPB, BT, JB and IM were responsible for conducting the experiments; CPB, PB, and KA did the statistical evaluations and drafted the manuscript. All authors revised the manuscript for important intellectual content and approved the final manuscript.

## Pre-publication history

The pre-publication history for this paper can be accessed here:

http://www.biomedcentral.com/1471-2369/11/31/prepub
